# Plant-Based Dietary Patterns and Cardiovascular Disease Risk in Australians: Protocol for a Cross-Sectional Study

**DOI:** 10.3390/nu15132850

**Published:** 2023-06-23

**Authors:** Jessica J. A. Ferguson, Grace Austin, Christopher Oldmeadow, Manohar L. Garg

**Affiliations:** 1 Nutraceuticals Research Program, School of Biomedical Sciences & Pharmacy, University of Newcastle, 305C Medical Science Building, Callaghan, NSW 2308, Australia; jessica.ferguson@uon.edu.au (J.J.A.F.); grace.austin@newcastle.edu.au (G.A.); 2 Clinical Research Design, Information Technology and Statistical Support Unit, Hunter Medical Research Institute, University of Newcastle, New Lambton, NSW 2308, Australia; christopher.oldmeadow@newcastle.edu.au

**Keywords:** plant-based diets, vegetarian, vegan, dietary patterns, diet, cardiovascular disease, CVD, cross-sectional, Australia

## Abstract

Plant-based diets (PBDs) emphasise higher intakes of plant foods and lower intakes of animal foods, and they have been associated with reduced cardiovascular morbidity/mortality and lower cardiovascular disease (CVD) risk factors. Evidence is limited regarding the dietary profile, diet quality, and nutritional adequacy of PBDs, including their impact on CVD risk compared with traditional meat-eating diets in Australians. The PBD Study (PBDS) is a cross-sectional study that will recruit 240 adults from the Hunter region (NSW) without known CVD who are habitually consuming vegan (no animal flesh/animal products), lacto-ovo vegetarian (dairy and/or eggs only), pesco-vegetarian (fish/seafood only), or semi-vegetarian (minimal animal flesh) diets or are a regular meat-eater. To investigate dietary profile, diet quality, nutritional adequacy, and CVD risk, questionnaires (medical history, demographics, and physical activity), blood samples (biomarkers), physical measures (anthropometry, blood pressure, body composition, and bone density), and dietary intake (food frequency questionnaire and diet history) will be collected. One-way ANOVA and Kruskal–Wallis tests will compare the CVD risk and other quantitative measures, and Chi-square or Fisher’s Exact tests will be used for qualitative data. Directed acyclic graphs will determine the confounding variables, and linear regression and mediation analyses will account for the confounders and estimate the effect of dietary patterns on CVD risk. *p*-values will be adjusted using the Benjamini–Hochberg method to control the False Discovery Rate to 5%.

## 1. Introduction

Plant-based diets (PBDs) focus on high intakes of plants/plant-based foods and low intake of animal flesh/animal-based products and have been gaining traction worldwide. The global movement towards a PBD has several drivers, such as ethical concerns [1], reduced environmental impact and improved sustainability of the food system [2,3,4], concerns for animal welfare [1], and perceived healthiness [1,5]. A recent systematic review and meta-analysis of 12 prospective cohort studies, which included more than 508,000 participants, reported an inverse association between PBDs and risk of all-cause mortality and coronary heart disease mortality [6]. Moreover, another meta-analysis of 18 cross-sectional and 10 prospective cohort studies demonstrated that adherence to vegetarian/vegan dietary patterns compared with omnivorous dietary patterns was associated with lower BMI, blood cholesterol, LDL-cholesterol, and glucose concentrations. A significantly lower risk of ischaemic heart disease and total cancer mortality was also evident in the vegetarian/vegan dietary patterns [7]. PBD indexes have enabled investigation into the degree of adherence to and quality of PBDs and their subsequent health outcomes, with healthy PBDs that are rich in wholegrains, fruits, vegetables, legumes, nuts, tea, and coffee and low in animal foods being associated with lower risk of cardiovascular disease (CVD) mortality and all-cause mortality [8]. Conversely, unhealthy PBDs are characterised by having a higher intake of total energy and energy from carbohydrates and a lower intake of fruits, vegetables, and, thus, lower fibre and micronutrient intake [8]. Individuals following these dietary patterns have been shown to be more likely to drink higher amounts of alcohol, be less physically active and obese, and have hypertension [8].

Despite the promising findings of the aforementioned literature, the majority of evidence around PBDs and their impact on health outcomes are reported in populations from USA and Europe [6,9], with only one cohort study to date conducted in Asia and one in Australia [10]. Market research has demonstrated that the adoption of vegetarian-style dietary patterns by Australians is rising from 1.7 million in 2012, to 2.2 million in 2014, and to 2.5 million in 2018 (more than 12% of the total population) [11]. Despite the growing popularity of PBDs among Australians, limited evidence exists around the dietary profile, nutritional adequacy, and chronic disease risk specific to the Australian population. The lack of standardised definitions and classification of PBDs has also been acknowledged [6] and requires refinement to examine and compare adequately the dietary intake, nutritional concerns, and health outcomes across the spectrum of PBDs compared with traditional meat-eating diets.

Secondary analysis of the ‘45 and Up’ study by the Sax Institute is the only cohort study to investigate the effect of vegetarian diets (including various categories of vegetarian diets) and all-cause mortality in Australians and, converse to studies conducted in other countries, found there to be no evidence that following a vegetarian style dietary pattern has an independent protective effect on all-cause mortality [10]. Although the longitudinal cohort study was large (*n* = 243,096), rigorously designed, and extensively controlled for confounders, several limitations pertain to the sole reliance on it for informing the PBD climate among Australians; these are (1) the study primarily aimed to investigate a wide range of exposures and outcomes of public health importance for the ageing population; (2) a 24 h recall or FFQ was not employed; thus, diet was examined only through brief dietary behaviour questions in the baseline questionnaire; (3) limited PBDs were examined, as researchers could not distinguish between vegans and lacto-ovo vegetarians; and (4) the study was conducted between 2006 and 2014. The ‘45 and Up’ study provides historical insight into vegetarian-style diets and their association with disease, but, moreover, it initiates the need for an Australian-population cohort study designed primarily to examine the current association between PBDs and human health.

Through the adaptation of dietary categorisations employed in the ‘45 and Up’ study, we have demonstrated that individuals following PBDs had significantly lower body weight, central obesity, and BMI compared with regular meat-eaters in more than 9000 women from the Australian Longitudinal Study of Women’s Health (ALSWH) [12]. Moreover, regular meat-eaters consuming meat daily or multiple times per day had significantly higher body weight, central obesity, and BMI compared with those consuming meat less than daily but at least 2 times per week. Our study demonstrated a dose-dependent association between frequency of weekly meat intake and body weight, central obesity, and BMI, even after adjusting for the confounders [12]. In this same cohort, a significantly lower prevalence of diabetes was reported in vegetarians compared with meat-eaters, and high-quantity meat consumers reported a significantly higher prevalence of impaired glucose tolerance (manuscript under review). The prevalence of individuals following PBDs in both the ‘45 and Up’ study and ALSWH was only ~2%, resulting in the pooling of some data for comparison with regular meat-eaters, which limits specific diet and health comparisons across the PBD continuum. Noteworthy, the ‘45 and Up’ and ALSWH studies collected data from 2006–2014 and 2013, respectively, prior to the surge in global popularity of these diets; therefore, it is crucial that the current dietary profile of the PBD is investigated in Australians.

Only secondary analyses of population-based data form the basis of our current understanding and evidence around PBDs and health in the Australian population, and these findings reflect the PBD climate from 8–16 years ago, prior to the rapid surge in its adoption. Therefore, an Australian study methodologically targeting PBDs is warranted to address the national and global concern, which is the implementation of sustainable dietary patterns for future food ecosystems.

This Australian-first population-based study to examine the dietary profile, health status, and CVD risk of individuals following various PBDs will be titled the Plant-Based Diet Study (PBDS). This study will be strengthened by the purposeful recruitment methodology guided by specific strict dietary criterion to capture true habits of dietary patterns, which is not logistically and financially feasible to examine via a randomized controlled trial. Outcomes of this study will provide a database for studying PBDs, aid in the design of future longitudinal studies, and inform current Australian dietary guidelines, which are lagging regarding guidance towards sustainable dietary patterns.

## 2. Materials and Methods

### 2.1. Study Design and Setting

The PBDS is a cross-sectional study conducted at the Nutraceuticals Research Program, School of Biomedical Sciences & Pharmacy, University of Newcastle, Callaghan NSW, Australia. Eligible participants (Table 1) will be invited for one timepoint of data collection to examine the dietary profile, diet quality, nutritional status, and risk of developing CVD in adults consuming various PBDs and regular meat-eating diets (RMDs). To enhance representativeness of the Australian adult population and to gain a true understanding of the characteristics of the dietary patterns of interest, every effort will be made to invite all eligible individuals to participate in the study, including those with chronic health conditions.

**Table 1:** Inclusion and exclusion criteria for the PBDS.

Inclusion Criteria	Exclusion Criteria
Adults ages 35–75 years.	Ages <35 and >75 years.
No history of CVD, such as myocardial infarction, coronary insufficiency, angina, ischaemic stroke, transient ischaemic attack, haemorrhagic stroke, peripheral artery disease, heart failure, or pacemaker implant.	Receiving treatment for CVDs.
Consuming one of the following dietary patterns in Table 2 for at least 6 months; (1) vegan, (2) lacto-ovo vegetarian, (3) semi-vegetarian, (4) pesco-vegetarian, and (5) regular meat-eaters.	Made significant changes to their dietary pattern, food choices, lifestyle and/or physical activity levels in the past 6 months (determined by an Accredited Practising Dietitian) and/or practicing in (or in the last 30 days) another research trial involving dietary and/or physical activity intervention.
	Pregnant/lactating.

### 2.2. Recruitment and Consent

Participants will be recruited from the community via public notice board flyers, word of mouth, or publicity generated by media outlets, e.g., newspaper articles, radio announcements, or social media networks/groups, detailing the PBDS. Potential participants are invited to contact study investigators via phone or in person to clarify details of the study. They will be provided with the participant information statement and consent form and must return the completed consent form, including signature and date, to the investigators as a mandatory requirement for enrolment in the study. Eligibility will be assessed by lead investigators by phone or in person using the study eligibility checklist. Individuals who participated in earlier studies at our research facility will also be invited to participate.

### 2.3. Study Regime

**Figure 1:**
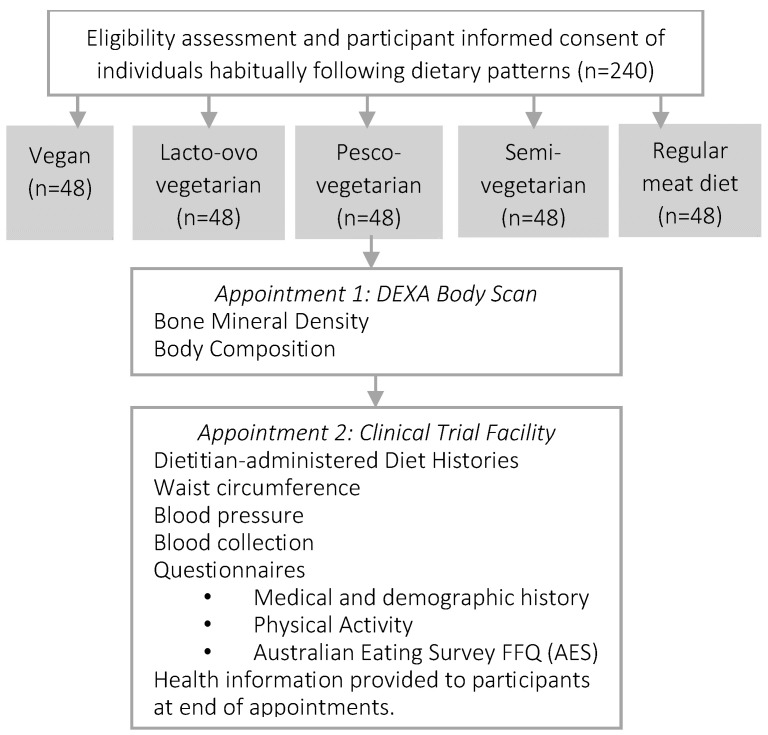
Flow diagram of the overall study design for the PBDS:DEXA, Dual X-ray absorptiometry scan; FFQ, food frequency questionnaire.

Data will be collected from consented enrolled participants once via two locations on the same day (or no more than 1–2 days apart) after an overnight fast (~10–12 h). First, body composition and bone density will be measured by a trained technician at the Newcastle Bone Density Centre (Waratah, NSW, Australia), and the remaining measurements and data will be collected at the Nutraceuticals Research Program at the University of Newcastle (Callaghan, NSW, Australia). These measurements will be conducted by the lead investigator and include seated blood pressure; waist circumference; diet history; medical, demographic, ethnicity, and physical activity history; and fasted blood samples for lipaemic parameters (total cholesterol, LDL-cholesterol, HDL-cholesterol, triglycerides, and total:HDL-cholesterol ratio), glycaemic parameters (glucose, insulin, and HbA1c), pro-inflammatory mediators (e.g., tumour necrosis factors alpha (TNF-α)), high-sensitivity C-reactive protein (hsCRP), full blood count, vitamin D, and liver function (Figure 1). Table 3 summarises data collected by participants unaided via self-administered questionnaires and data collected with assistance of a study investigator. All questionnaires have been either used in routine clinical care or were previously validated, and references are included within the tables, where appropriate. This cross-sectional study collects information from a singular time-point. Participants will be asked to provide consent to be contacted for potential future research.

The data will be stored on the University of Newcastle’s secure cloud-based system, which is suitable to host files that have large data or computing requirements. All electronic files will be password-protected as will be any study computers or laptops used for data entry in the first instance. The lead study investigator will enter the data, and double data entry and random cross-validation methods on the dataset will be routinely implemented to ensure validity, accuracy, and quality of the data.

### 2.4. Primary Outcomes

The primary outcome of this study is the 10-year and 5-year risk of developing CVD.

The Framingham Risk Equation (2008) [15] is a single multivariable risk function that will be used to calculate the 10-year risk of developing atherosclerotic CVDs defined as coronary death, myocardial infarction, coronary insufficiency, angina, ischaemic stroke, transient ischaemic attack, haemorrhagic stroke, peripheral artery disease, and heart failure. This equation was developed from the long-running Framingham Heart Study and is targeted at individuals aged 30 to 74 years of age without CVD at baseline examination [16]. There are two versions of the equation; the version which uses lipids will be used for this study. Variables that are required for the equation include (1) sex; (2) age; (3) total cholesterol; (4) HDL-cholesterol; (5) systolic blood pressure; (6) treatment status for hypertension; (7) smoking status; and (8) diabetes status. The Framingham Risk Equation has been validated in non-Indigenous Australian adults, with the 2008 equation reported to perform better than previous Framingham Risk equations at predicting cardiovascular outcomes in relatively young, healthy Australians. Therefore, the 2008 Framingham Risk Equation will be used for this study [17].

The 5-year absolute CVD risk will be estimated using the Australian Absolute CVD Risk Calculator [18] developed by the National Stroke Foundation on behalf of the National Vascular Disease Prevention Alliance (NVDPA), now part of the Australian Chronic Disease Prevention Alliance (ACDPA), with partner agencies and expert clinicians [19]. The Australian Absolute CVD Risk Calculator is also based on the Framingham Risk Equation. Descriptors of risk categories in the Australian context will be defined as in the Guidelines for the Assessment of Absolute Cardiovascular Disease Risk (2009) [19,20] and are presented in Table 4.

### 2.5. Secondary Outcomes

This study has the following secondary outcomes to investigate across various PBD and regular meat-eating diets: (1) dietary profile, such as dietary intake and food choices, across the five food groups; (2) diet quality and nutritional adequacy; (3) body composition and anthropometric measures; (4) bone mineral density; (5) biochemical parameters; and (6) medical, demographic, lifestyle, and physical activity characteristics. Secondary analyses of duration of following dietary pattern may be explored using a dose–response analysis, with the number of years being the exposure variable. Only sex would be adjusted for, as other variables are influenced by time.

### 2.6. Clinical Assessments and Laboratory Investigations

#### 2.6.1. Blood Sampling and Analyses

Fasted blood samples will be collected via venepuncture into Vacuette^®^ blood tubes by an experienced phlebotomist. Samples will be centrifuged (Heraeus Biofuge Stratos, Germany, Heidelberg, sourced from Australia) for 15 min at 3000× *g* at 4 °C. Plasma, red blood cells, and buffy coat fractions will be aliquoted and stored at −80 °C until further analysis. Biochemical parameters, such as lipids (total cholesterol, LDL-cholesterol, HDL-cholesterol, triglycerides, and total-cholesterol-to-HDL-cholesterol ratio); glycaemic indices (glucose, insulin, and HbA1c); vitamin D (25-hydroxy vitamin D); full blood count; liver function, and high-sensitive C-reactive protein will be measured on a VP auto analyser using standardized reagents by the commercial pathology service provider NSW Health Pathology–North. Pro-inflammatory mediators, such as interleukin, tumour necrosis factor α, and intercellular adhesion molecule-1, will be quantified by enzyme-linked immunosorbent assay as per the manufacturer’s instructions (Abcam, Cambridge, UK) at the Nutraceuticals Research Program, University of Newcastle, (Callaghan, NSW, Australia). Polygenic risk scores will be measured to predict the likelihood of specific outcomes by including DNA variants in a statistical model.

#### 2.6.2. Anthropometry and Blood Pressure

Height will be measured using a wall-mounted stadiometer with a movable head piece (Seca 206 Bodymeter Wall Height Measure Ruler, Hamburg, Germany, sourced from Australia). Height (cm) and weight (kg) will be collected to the nearest 0.1 units in light clothing without shoes. A tensible tape measure positioned midway between the lower rib margin and the iliac crest (approximately in line with the bellybutton) horizontally will be used to measure waist circumference (cm). Blood pressure will be measured in the seated position, and three serial measurements with 1-min rests in between will be collected from a supported left arm of a rested participant. The first measurement will be discarded, and an average of the remaining two will be considered as the final measurement.

#### 2.6.3. Body Composition

Body mass and composition will be measured via dual-energy X-ray absorptiometry (DXA) using the GE Lunar Prodigy DXA machine (narrow angle fan beam technology) at the Newcastle Bone Density Centre, which is a private medical imaging centre in Newcastle. Body mass (kg), fat mass (kg), fat percentage, fat mass index, lean muscle mass (kg), lean muscle mass percentage, lean muscle mass index, body mass index (kg/m^2^), android/gynoid fat tissue distribution (% and ratio), waist-to-hip ratio, relative skeletal muscle index (kg/m^2^), bone mass (kg), and bone mineral density (g/cm^2^) will be quantified. At the Newcastle Bone Density Centre, all scans will be performed by qualified and experienced clinicians. Participants will attend the centre once, either on the same day or no more than 1–2 days of their clinic appointment at the University of Newcastle.

#### 2.6.4. Dietary Assessment

The Australian Eating Survey^®^ (AES) is a self-administered, validated, and reliable FFQ used to measure usual food and nutrient intake over the past 3 to 6 months [14]. The AES will be used to categorise dietary patterns, investigate the dietary profile of various dietary patterns, and assess diet quality and nutrient intake. The AES FFQ uses a subset of 70 questions related to core nutrient-dense foods recommended in the Australian Dietary Guidelines [23] and has been evaluated in Australian populations from the age of two years and is based on 15 years of research [14]. The Australian Recommended Food Score (ARFS) will be used to investigate diet quality across dietary patterns groups. The ARFS is derived from the AES FFQ by summing the points within eight sub-scales, as previously reported [24]. A comprehensive diet history capturing habitual food, beverage, and supplement intake for two typical days will also be collected during the appointment with the study coordinator who is also an Accredited Practising Dietitian and Accredited Nutritionist. An average of nutrient intake across the two days will be used to confirm findings from the FFQ, including directive questioning around motivations towards dietary patterns, such as *‘How long have you been consuming this type of dietary pattern?*’, ‘*What made you commence eating this type of dietary pattern?’,* and, when relevant, *‘What dietary pattern where you previously following?’* Other characteristics around dietary patterns will also be summarised, for example, frequency of consumption of dairy alternatives, plant-based meat alternatives, and their fortification status. Dietary pattern data from the AES and diet histories will be compared against the current 2013 Australian Dietary Guidelines (qualitatively) [23], and macronutrient and micronutrient data will be compared against the Nutrient Reference Values for Australia and New Zealand (quantitatively) [25]. Dietary intakes assessed from diet histories will be summarised and quantified using FoodWorks (Xyris^®^, Brisbane, Australia, sourced online).

#### 2.6.5. Medical History, Demographics, and Physical Activity

A self-administered medical history and demographic questionnaire will collect information regarding past and present medical conditions, including family history of CVDs; prescribed or over-the-counter medication(s); habitual supplement use; habitual consumption of alcohol; ethnicity; marital and occupation status; and level of education and income. Data required for the CVD risk prediction models will also be collected from the medical history and demographic questionnaire, such as diabetes status (defined as current treatment with oral anti-diabetic medications or insulin and confirmed by reported diagnosis from the participant’s general practitioner), smoking status, and report of antihypertensive medication prescribed for high blood pressure/hypertension. The International Physical Activity Questionnaire (IPAQ, Long Version October 2002) is a self-administered and validated questionnaire that asks participants to reflect on their physical activity levels in the last 7 days [13]. This will be used to assess habitual physical activity levels. Physical activity data will be interpreted as metabolic equivalent of task minutes per week (MET/week) to measure the energy cost of physical activities.

### 2.7. Sample Size

Based on previous estimates of variance in 10-year CVD risk scores using the Framingham algorithm (Mean = 13.3, SD ± 5.77) [26] to elicit 80% power at a significance level of 0.05 to detect a 25% (Δ = 3.33) difference in CVD risk scores among at least one group following a PBD pattern compared with a regular meat-eating dietary pattern, a sample size of *n* = 240 participants (48 per dietary pattern) is required. Previous observational studies on dietary pattern and lifestyle have reported differences of 27% and 20–22% in Framingham Risk Scores [27,28]. Moreover, we have reported reductions of 13–23% in RCTs following dietary interventions for only 3–4 weeks [29,30]. A longitudinal study which employed a healthy PBD index reported a 47–68% lower risk of developing CVD in individuals among the 2nd and 3rd tertiles of adherence to a healthful PBD compared with their 1st tertile counterparts [31]. Similarly, vegetarians have been shown to have a 22% lower rate of ischaemic heart disease than meat-eaters in the EPIC-Oxford study of more than 48,000 participants followed up after 18 years [32]. Therefore, after review of previous studies’ CVD risk outcomes, the authors arrived at the 25% estimated difference in Framingham Risk Score between at least the two extreme dietary patterns (vegan vs. regular meat-eating diet). This cross-sectional study meets the target sample size of 48 per group to be statistically powered, and estimates are substantially supported by the previous literature. Authors recognise that the modest sample size of 240 may be used as a pilot or ‘proof of concept’ study for larger cohorts in the future.

### 2.8. Statistical Analyses

**Figure 2:**
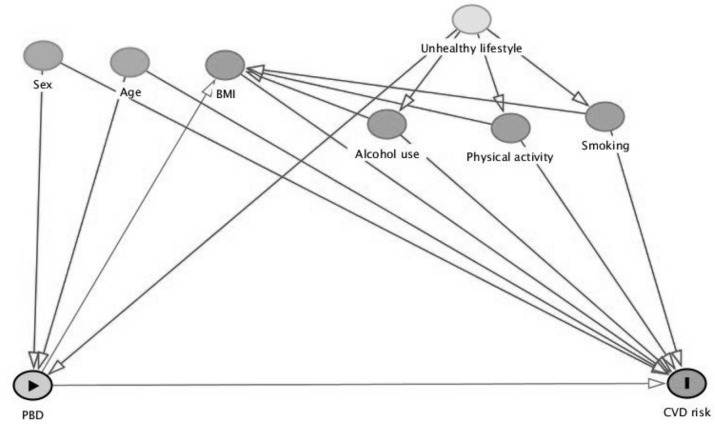
Directed acyclic graph showing independence among potential confounding and mediating variables on the association between PBDs and CVD risk. Confounding factors are age, sex, alcohol use, smoking status, and physical activity levels, and BMI is a mediator. BMI, body mass index; PBDs, plant-based diets; CVD, cardiovascular disease.

All data will be assessed for normality by inspecting visual plots, such as histograms, quantile plots, and Q-Q plots, within each dietary pattern group for each variable. One-way ANOVA or Kruskal–Wallis tests will be used for comparison of CVD risk scores, dietary intake, and cardiometabolic parameters, including all clinical measures, medical/demographics, and blood parameters across dietary pattern groups. Chi-square or Fisher’s Exact tests will be used to compare qualitative dietary intake data and other categorical data across dietary pattern groups. Directed acyclic graphs (DAGs) will be used to display dependencies between outcome (5-year absolute CVD risk and 10-year CVD risk scores), exposure (different categories of dietary patterns determined via classifications presented in Table 1), and confounding/mediating variables to determine the minimum set of variables necessary to control for confounding [33] (Figure 2). Linear regression will be used to account for potential confounding factors described in the DAGs, such as age, sex, alcohol use, smoking status, and physical activity levels. Mediation analysis will be used to estimate the effect of PBDs on CVD risk that is not mediated through BMI [33,34]. Predictably, there will be intrinsic differences between dietary pattern groups, as the study design endeavours to evaluate the true characteristic of each group (chronic diseases, lifestyle choices, etc.). To address this and remove risk of overadjustment, a sensitivity analysis will be performed on potential additional important confounding variables not already identified in the DAGs to assess how robust the results are when comparting the additional confounding factor [35,36]. *p*-values will be adjusted using the Benjamini–Hochberg method to control the False Discovery Rate to 5%.

All statistical analyses will be conducted using StataCorp. 2015. *Stata Statistical Software: Release 14* (College Station, TX, USA: StataCorp LP). Continuous data will be presented as means, SD, median, and interquartile range (25th–75th percentile), and categorical data will be summarised as frequencies and percentages.

### 2.9. Sources of Bias

Major sources of bias in this protocol relate to selection bias and self-reported data, both of which are inherent sources of bias in observational studies [37,38]. We acknowledge that individuals volunteering to participate in the study (as in most clinical studies) may be from a pool of participants who might be better educated, of higher socioeconomic status, or health-motivated [37]. This protocol aims to employ analytical strategies to summarise and adjust for various health and demographic characteristics and has been reported in the statistical section. Like many other cross-sectional/observational studies, this protocol relies on a significant amount of self-reported data from participants. This introduces an inherent source of bias in self-collected data, and it cannot be avoided [38]. Researchers will thoroughly check all self-collected data and revise hardcopy and electronically submitted questionnaires with participants to ensure correct interpretation of questionnaires and that correct data have been entered by participants.

### 2.10. Ethics and Dissemination

#### 2.10.1. Ethics

This study has been approved by the University of Newcastle’s Human Research Ethics Committee (HREC 2020-0195). Eligibility will be assessed by lead investigators by phone or in person using the eligibility checklist. Eligible volunteers will be provided with the information statement, and written informed consent will be obtained. Findings will be reported to PBDS participants; funding bodies and institutes; and federal, state, and local governments to inform policy (should opportunity arise); findings will be presented at local, national, and international conferences and disseminated by peer-reviewed publications.

#### 2.10.2. Participant Safety

All risks to the participants in the study will be mitigated by ensuring recruitment and data collection are managed by appropriately trained and experienced research investigators with all the required certifications and qualifications. Recruitment will be undertaken by Accredited Practising Dietitians with extensive experience in clinical research recruitment and dietary interventions to ensure accurate and thorough conduct of dietary screening and eligibility. Data will be stored in a secure setting, with password-protected devices and cloud storage systems. All participants are required to complete a DXA Safety Screening Questionnaire on the day of their DXA scan, and all scans will be performed by trained technicians according to standard operating procedures by the external service provider, Newcastle Bone Density Centre. A dosimetry report by a medical physicist has also been obtained for this protocol, deeming radiation exposure associated with this study to be safe with negligible risk. A trained phlebotomist will conduct all venepuncture blood sample collections under standard operating procedures.

#### 2.10.3. Unexpected Findings during Examinations

At the end of the study appointment, participants will be provided with their personal study results, i.e., body composition output, blood pressure, and FFQ survey results outlining food and nutrient intake in accordance with national recommendations. Routine blood test results analysed by the service provider will be provided to participants as soon as they are received by the researchers via the participants nominated preferred method of contact, e.g., email or post. All participants will be encouraged to visit their General Practitioner should they have any questions or concerns regarding any of their personal results. The researchers will not provide any health advice or recommendations based on clinical findings from this study.

#### 2.10.4. Dissemination

The findings from analysis of the PBDS will be disseminated in various platforms, such as manuscripts in peer-reviewed journals and scientific conference abstracts, presentations, posters, and a higher degree research thesis. These will also be reported to local, state, and federal governments to inform policy and new and revised national dietary guidelines. Findings from this cross-sectional study may inform the development of specific dietary guidelines for PBDs as well as inform current dietary guidelines related to cardiovascular health. Reports made to funding bodies and institutes that supported the PBDS will also be made from these study findings. Members of the research team will have publishing and authorship rights in accordance with National Health and Medical Research Council Australian Code for the Responsible Conduct of Research and as described in research agreements.

## 3. Discussion

The PBDS is an Australian-first cross-sectional study exploring the cardiovascular health and dietary profile of PBDs. The novelty of this cross-sectional study pertains to the methodological design of purposeful recruitment of habitual consumers of various PBDs and meat-eating diets. Previous studies examining the link between PBDs and cardiometabolic health have been secondary analyses in cohorts not primarily designed to investigate the association between PBDs and cardiometabolic diseases. Moreover, myriad different terms and characteristics have been used when defining various PBDs, leading to discrepancies and possibly to misclassification. The globe’s growing adoption of sustainable dietary patterns alongside major health authorities, such as the World Health Organization (WHO) and the Food and Agriculture Organization of the United Nations (FAO), emphasising plant-forward eating highlights the critical need for population-based evidence to guide the safe and effective implementation of these dietary patterns in Australia. This work will identify the dietary profile and nutrition implications of various PBDs currently adopted by Australians alongside their impact on cardiometabolic risk factors. This study will provide a database for future cross-sectional investigations exploring characteristics of PBD in Australia and linking PBD and health. Moreover, this work will serve as a body of evidence with which to develop a well-defined longitudinal study to examine the association between PBDs and the development of chronic diseases over time.

There is a need to establish population-specific, appropriate, and up-to-date cardiovascular risk prediction equations for Australians [17]. The absolute risk equation (based on the Framingham Risk Equations) currently used in Australia may overestimate risk in individuals under 30 years of age [19] and underestimate CVD risk in specific populations, such as Aboriginal and Torres Strait Islander peoples, South Asian, Māori, and Pacific Islanders, and Middle Eastern persons; adults with diabetes; adults aged over 74 years adults with depression; and adults who are socioeconomically disadvantaged [19]. To mitigate this, the current study will not include individuals aged under 30 years nor those over 70 years, and a large breadth of additional CVD risk factors and lifestyle characteristics/behaviours will be collected from the participants’ medical history: physical measures, including body composition, biochemical parameters, socio-economic and demographic characteristics, and dietary intake. Investigation of these significant risk factors that are not currently included in the risk equations will provide extra predictive value and inform the overall interpretation of the risk of developing CVD in association with dietary patterns explored in this study.

The novel outcomes of the PBDS include the following: (1) investigation of the 5-year and 10-year risk of developing CVD across the spectrum of PBDs and meat-eating dietary pattern; (2) analysis of biological samples to understand the biological mechanisms driving cardiometabolic health outcomes/risk across dietary patterns; (3) investigation and analysis of diet intake, quality, and nutritional adequacy across the spectrum of PBD and meat-eating dietary patterns; (4) development of rich data that characterise the health, socio-economic, behaviour, and medical history of individuals following PBD and meat-eating dietary patterns to improve the understanding of the interplay between PBDs and CVD risk; and (5) implementation of standardised definitions of a PBD in the Australian population in an epidemiological setting. To our knowledge, there is no other cross-sectional study within Australia or internationally where all these five points are explored in a single study with this design.

The PBDS commenced recruitment on 10 November 2021, and despite having some delay due to the COVID-19 pandemic, we have been able to recruit individuals from the community, with several participants already enrolled into each of the dietary pattern categories. The study remains in the recruitment and data collection phase until at least mid-2023 before data cleaning, coding, and analyses will commence. The study is expected to be completed by mid-2024. As with many observational studies, some of the data collected in this study are self-reported; however, several of the data collection tools employed in this study have been validated in Australian populations. To further mitigate any risk associated with self-reported data, all data are cross-checked by study researchers and clarified (when required) with participants to their ensure accuracy and the correct interpretation of the questionnaires or instructions. As with all cross-sectional population-based studies, caution must be exercised when inferring causation from the results. As detailed in the protocol, appropriate statistical analyses will be conducted to summarise and adjust for the confounding factors and covariates, and a senior biostatistician will be involved in the project.

To conclude, given several strengths and the significance of this study’s findings, albeit with some limitations, analysis of the data arising from this study will likely influence nutrition policy and dietetic practice around the influence of ‘plant-forward’ eating patterns for heart health.

## Figures and Tables

**Table 2:** Classification of dietary pattern groups by the number of time(s) foods are consumed, on average, per week.

	Vegan	Lacto-Ovo Vegetarian	Semi-Vegetarian	Pesco-Vegetarian	Regular Meat-Eater
Times per week consumed:					
Red meats:	0	0	≤1	0	0 or ≥1
Beef, kangaroo, veal, lamb, pork, etc.					
White meats:	0	0	≤1	0	0 or ≥1
Chicken, turkey, duck, etc.					
Processed/cured meats:	0	0	≤1	0	0 or ≥1
Bacon, corned beef, luncheon meats, such as ham, prosciutto, salami, etc., sausages, frankfurters					
*Seafood:* Fish (including tinned), crustaceans, oysters, squid, mussels, etc.	0	0	0 or ≤1	≥1	0 or ≥1
**Total of above categories**	0	0	≤2	≥1	≥7
Usual eating habits					
Animal-based dairy products (milk, cheese, ice-cream, butter, cream, margarine blends, etc.)	Nil	Y	N/A	N/A	N/A
Eggs	Nil	Y	N/A	N/A	N/A

Defining characteristics of dietary patterns are adapted from those previously implemented in Australian cohorts by Ferguson et al. [12] and Mihrshahi et al. [10]. ‘N/A’ denotes characteristics that were not relevant and, thus, not used for categorizing into respective diet groups.

**Table 3:** Questionnaires, histories, measures, and biochemical samples completed by participants unaided and with assistance of a study investigator.

Item	Measures and Tools	Collected by Researcher or Participant ^1^
Medical history, medication/supplement use, and family medical history	Medical and Demographic Questionnaire	Participant
Demographics, ethnicity, socio-economic status, education history, employment history, smoking history, habitual alcohol intake, marital status, household food, and social history	Medical and Demographic Questionnaire	Participant
Physical activity	International Physical Activity Questionnaire (IPAQ-Long Form 2002) [13]	Participant
Dietary intake (qualitative), diet quality	Australian Eating Survey Food Frequency Questionnaire (AES FFQ) [14]	Participant
Food and nutrient intake (quantitative) and dietary pattern characteristics	Diet history via Accredited Practicing Dietitian	Researcher
Fasted biochemical parameters	Venepuncture blood sample	Researcher
Blood pressure and waistline	Seated systolic and diastolic blood pressures Waist circumference	Researcher
Height and body composition	Dual X-ray absorptiometry scan	Researcher

^1^ Collected by participant via self-administered questionnaire.

**Table 4:** Qualitative risk categories used to describe calculated 5-year absolute CVD risk using the Australian Absolute CVD Risk Calculator and 10-year CVD risk using the Framingham Risk Equation.

Risk Category	5-Year CVD Risk Description ^1^	10-Year CVD Risk Description ^2^
**Low risk**	<10% probability of CVD within the next five years	<10% probability of CVD within the next ten years
**Moderate/Intermediate risk**	10–15% probability of CVD within the next five years	10–20% probability of CVD within the next ten years
**High risk**	>15% probability of CVD within the next five years	>20% probability of CVD within the next ten years

^1^ Guidelines for the assessment of absolute CVD risk [20]. ^2^ Categories of absolute CVD risk percentage over 10 years, classified according to previous studies [21,22].

## Data Availability

Data from the PBDS will be made available in the future for collaborative research questions. Such requests must be authorised by the principal investigators and the appropriate Human Research Ethics Committees.
